# Long noncoding RNA NEAT1 drives aggressive endometrial cancer progression via miR-361-regulated networks involving STAT3 and tumor microenvironment-related genes

**DOI:** 10.1186/s13046-019-1306-9

**Published:** 2019-07-08

**Authors:** Peixin Dong, Ying Xiong, Junming Yue, Daozhi Xu, Kei Ihira, Yosuke Konno, Noriko Kobayashi, Yukiharu Todo, Hidemichi Watari

**Affiliations:** 1 Department of Obstetrics and Gynecology, Hokkaido University School of Medicine, Hokkaido University, Sapporo, Japan; 2 Department of Gynecology, State Key Laboratory of Oncology in South China, Sun Yat-sen University Cancer Center, Guangzhou, People’s Republic of China; 3 Department of Pathology and Laboratory Medicine, University of Tennessee Health Science Center, Memphis, TN 38163 USA; 4 Center for Cancer Research, University of Tennessee Health Science Center, Memphis, TN 38163 USA; 5 Division of Gynecologic Oncology, National Hospital Organization, Hokkaido Cancer Center, Sapporo, Japan

**Keywords:** NEAT1, miR-361, STAT3, Endometrial cancer metastasis, Tumor microenvironment

## Abstract

**Background:**

High-grade endometrioid and serous endometrial cancers (ECs) are an aggressive subtype of ECs without effective therapies. The reciprocal communication between tumor cells and their surrounding microenvironment drives tumor progression. Long noncoding RNAs (lncRNAs) are key mediators of tumorigenesis and metastasis. However, little is known about the role of lncRNAs in aggressive EC progression and tumor microenvironment remodeling.

**Methods:**

We performed an array-based lncRNA analysis of a parental HEC-50 EC cell population and derivatives with highly invasive, sphere-forming, and paclitaxel (TX)-resistant characteristics. We characterized the roles of the lncRNA NEAT1 in mediating aggressive EC progression in vitro and in vivo and explored the molecular events downstream of NEAT1.

**Results:**

We identified 10 lncRNAs with upregulated expression (NEAT1, H19, PVT1, UCA1, MIR7-3HG, SNHG16, HULC, RMST, BCAR4 and LINC00152) and 10 lncRNAs with downregulated expression (MEG3, GAS5, DIO3OS, MIR155HG, LINC00261, FENDRR, MIAT, TMEM161B-AS1, HAND2-AS1 and NBR2) in the highly invasive, sphere-forming and TX-resistant derivatives. NEAT1 expression was markedly upregulated in early-stage EC tissue samples, and high NEAT1 expression predicted a poor prognosis. Inhibiting NEAT1 expression with small hairpin RNAs (shRNAs) diminished cellular proliferation, invasion, sphere formation, and xenograft tumor growth and improved TX response in aggressive EC cells. We showed that NEAT1 functions as an oncogenic sponge for the tumor suppressor microRNA-361 (miR-361), which suppresses proliferation, invasion, sphere formation and TX resistance by directly targeting the oncogene STAT3. Furthermore, miR-361 also suppressed the expression of multiple prometastatic genes and tumor microenvironment-related genes, including *MEF2D*, *ROCK1*, *WNT7A*, *VEGF-A*, *PDE4B,* and *KPNA4*.

**Conclusions:**

NEAT1 initiates a miR-361-mediated network to drive aggressive EC progression. These data support a rationale for inhibiting NEAT1 signaling as a potential therapeutic strategy for overcoming aggressive EC progression and chemoresistance.

## Background

Endometrial cancer (EC) is the most frequently diagnosed gynecological cancer in developed countries and the second leading cause of gynecological cancer-related death among females [1]. Unlike grade 1–2 endometrioid ECs (comprising ~ 80% of all ECs diagnosed), which have more favorable patient outcomes, grade 3 endometrioid and serous ECs are more biologically aggressive variants of EC and are resistant to currently available treatments [2]. A better understanding of the molecular mechanisms underlying cancer initiation, progression, and resistance acquisition would aid in developing effective therapeutic strategies for aggressive EC.

Tumor progression is a complex process that involves tumor cell proliferation, detachment, invasiveness, blood and lymphatic vessel entry, and eventual extravasation at distant sites [3]. Tumor progression is closely associated with extracellular matrix remodeling, angiogenesis, inflammation and immune evasion [3]. Cancer stem cells (CSCs) have self-renewal abilities, differentiate into heterogeneous lineages of cancer cells, and are considered to be responsible for cancer metastasis, cancer relapse and drug resistance [4]. Thus, there ere is an urgent need to identify critical molecular events that regulate the growth, invasion, and chemoresistance of aggressive ECs.

Long noncoding RNAs (lncRNAs) are regulatory RNA transcripts longer than 200 nucleotides with no or limited protein-coding capacity [5]. LncRNAs can act as guides, scaffolds, and molecular sponges in interactions with proteins, messenger RNAs (mRNAs) and microRNAs (miRNAs), leading to the formation of complex networks that play crucial roles in modulating various cancer phenotypes [6]. The lncRNA NEAT1 promotes many tumors by functioning as an endogenous competing RNA (ceRNA) for tumor suppressor miRNAs [7], but whether NEAT1 sponges miRNAs to drive aggressive EC progression and chemoresistance remains unclear.

To elucidate the exact roles and underlying mechanisms of NEAT1 in aggressive ECs, we used the representative aggressive EC cell line HEC-50 to generate derivatives with high invasiveness, sphere-forming ability and paclitaxel (TX) resistance. Knocking down NEAT1 expression attenuated cellular proliferation, invasion, and sphere formation and sensitized aggressive EC cells to TX treatment. Mechanistically, NEAT1 serves as a molecular sponge for miR-361, a pivotal tumor suppressor that suppresses proliferation, invasion, sphere formation and TX resistance by directly targeting the oncogene STAT3. We further showed that miR-361 downregulates the expression of multiple prometastatic, proangiogenic and inflammation-related genes. Our findings identified an essential NEAT1-miR-361 axis that contributes to aggressive EC progression and chemoresistance, producing potential translational implications for retarding aggressive EC progression and improving the efficiency of chemotherapy for aggressive EC.

## Materials and methods

### Cell culture and functional assays

The human EC cell line HEC-50 (JCRB Cell Bank, Osaka, Japan) was derived from poorly differentiated endometrioid EC and cultured in DMEM/F12 (Sigma-Aldrich, St. Louis, MO, USA) supplemented with 10% fetal bovine serum (FBS). The human serous EC cell line SPAC-1-L was obtained from Dr. Fumihiko Suzuki (Tohoku University, Sendai, Japan) and maintained in RPMI-1640 medium (Sigma-Aldrich, St. Louis, MO, USA) supplemented with 10% FBS. The immortalized human endometrial epithelial cell line EM was kindly provided by Dr. Satoru Kyo (Shimane University, Japan) and grown in DMEM/F12 supplemented with 15% FBS. The STAT3 inhibitor BP-1-102 was purchased from Merck Millipore (Germany).

Highly invasive subpopulations of HEC-50 cells (referred to as HI cells) have been described previously [8]. We used serial sphere culture to enrich sphere-forming cells from the parental cell line HEC-50 as previously reported [9]. In brief, HEC-50 cells were grown under serum-free, nonadherent conditions. Following 14 days of culture, ball-like spheres were observed. These spheres were dissociated into single cells, and this process was repeated 3 times to enrich the sphere-forming cells (referred to as HEC-50-sphere cells). TX-resistant derivatives of HEC-50 cells were established by exposure to increasing concentrations of TX (Cell Signaling Technology, Beverly, MA), and the surviving resistant daughter cells were designated HEC-50-TX cells. The increase in TX resistance, known as fold resistance, was determined as the ratio of the 50% inhibitory concentration (IC50) of the invasive, sphere-forming derivatives and HEC-50-TX cells to the IC50 of the parental HEC-50 cells. Cells were transfected using Lipofectamine 2000 (Invitrogen, Carlsbad, CA) according to the manufacturer’s instructions. Cell proliferation, invasion and sphere formation were investigated by colony formation, Matrigel invasion, and sphere formation assays, respectively, as described previously [9].

### Construction of stable NEAT1 expression knockdown cell lines

Two short hairpin RNAs (shRNAs) targeting NEAT1_2 (NR_131012.1, the longer transcript of NEAT1) were designed using the web-based software siRNA Wizard (www.sirnawizard.com; InvivoGen, San Diego, CA, USA) and cloned into the BbsI sites of the psiRNA-h7SKneo G1 expression vector (InvivoGen). The NEAT1 shRNA-1 sequences were as follows: Oligo 1, 5′-ACCTCGATGCCACAACGCAGATTGATTCAAGAGATCAATCTGCGTTGTGGCATCTT-3′ and Oligo 2, 5′-CAAAAAGATGCCACAACGCAGATTGATCTCTTGAATCAATCTGCGTTGTGGCATCG-3′. The NEAT1 shRNA-2 sequences were as follows: Oligo 1, 5′- ACCTCGAAGATCCCTAAGCTGTAGAATCAAGAGTTCTACAGCTTAGGGATCTTCTT-3′ and Oligo 2, 5′-CAAAAAGAAGATCCCTAAGCTGTAGAACTCTTGATTCTACAGCTTAGGGATCTTCG-3′. A premade plasmid DNA vector (InvivoGen) expressing shRNA for enhanced green fluorescent protein (EGFP) was used as a negative control. HEC-50 cells were transfected with the above DNA plasmids and selected with G418 (Sigma-Aldrich, St. Louis, MO, USA) for 4 weeks.

### Total RNA extraction and qRT-PCR analysis

Total RNA was isolated from cells or tumor xenografts using TRIzol reagent (Invitrogen, Carlsbad, CA, USA) and subjected to reverse transcription using a Reverse Transcription Kit (Takara, Japan). The mRNA levels were determined using Takara SYBR Premix Ex Taq II (Takara, Japan) and an ABI7300 PCR instrument. For mRNA detection, all primers were obtained from the PrimerBank database (http://pga.mgh.harvard.edu/primerbank/). The PCR primers for lncRNAs were designed using Primer-BLAST (https://www.ncbi.nlm.nih.gov/tools/primer-blast/). The expression of mature miRNAs was measured using NCode miRNA qRT-PCR analysis (Invitrogen, Carlsbad, CA). The forward primers for miRNA detection had the same sequences as the mature miRNAs. The results were normalized to the levels of GAPDH for mRNA and lncRNA measurement or the levels of U6 for miRNA detection.

### LncRNA microarray profiling

Total RNA was extracted from cells using TRIzol reagent (Invitrogen, Carlsbad, CA, USA) and purified with an RNeasy Mini Kit (Qiagen, Valencia, CA, USA). RNA samples were subjected to the human lncRNA microarray V3 (Arraystar Inc., Rockville, MD, USA), which includes 30,600 lncRNA probes. The Agilent Array platform was used for the microarray analysis. Sample preparation, microarray hybridization, slide washing and scanning were performed according to standard Arraystar protocols. Agilent Feature Extraction software (version 11.0.1.1, Agilent Technologies) was used to analyze the acquired array images. Subsequent data processing was performed with the GeneSpring GX v11.5 software package (Agilent Technologies). Differentially expressed lncRNAs were identified with the criteria of log2 (fold change) > 2 and *P* < 0.01.

### Cell viability assay

Cells (5 × 10^3^) were plated in 96-well plates for 24 h and then treated with dimethyl sulfoxide (DMSO) or varying doses of TX (Cell Signaling Technology, Beverly, MA). After 24 h, cell viability was assessed using a Cell Counting Kit-8 assay (Dojindo, Kumamoto, Japan). Absorbance was determined at 450 nm using a microplate reader, and relative survival was calculated by comparing TX-treated cells with DMSO-treated cells.

### Tumor xenografts

For tumorigenesis assessment, female BALB/c-nu mice (aged 4 weeks) were purchased from Guangdong Medical Laboratory Animal Center (Guangzhou, China). All mice were maintained under specific pathogen-free conditions, and experimental procedures were performed according to the institutional animal welfare guidelines approved by the Institutional Animal Care and Use Committee of Sun Yat-Sen University Cancer Center. As described previously [10, 11], EC cells (2 × 10^6^) were injected subcutaneously into the flanks of nude mice under sterile conditions. Xenograft tumor formation was monitored using the following formula: tumor volume (mm^3^) = width (mm^2^) × length (mm) × 0.5. After 24 days, all mice were sacrificed, and the tumors were harvested for RNA isolation.

To determine the in vivo antitumor activity of TX, EC cells (2 × 10^6^) were injected subcutaneously into the flanks of nude mice. After the formation of palpable tumors, the mice were divided randomly into two groups, saline and TX groups, and treated via intraperitoneal injection twice a week for 24 days. Then, the tumors were weighed and analyzed by immunohistochemistry. Cell proliferation was measured in paraffin-embedded tumor tissue with immunohistochemical (IHC) staining using an anti-Ki-67 antibody (Abcam, Cambridge, UK).

### MiRNA microarray profiling

Total RNA was isolated from SPAC-1-L cells with NEAT1 expression knockdown and control cells using TRIzol reagent (Invitrogen, Carlsbad, CA, USA). Microarray analysis was performed using the Superprint G3 Human GE 8 × 60 k Microarray (Agilent Technologies) as described previously [12]. Volcano plot filtering was used to distinguish differentially expressed miRNAs with the criteria of log2 (fold change) > 2 and *P* < 0.01.

### Luciferase reporter assay

A wild-type (WT) or mutant (MUT) human NEAT1 fragment and the *MEF2D* 3′-UTR containing the miR-361 binding site were synthesized and cloned into the pGL3-basic vector (Promega, Madison, WI, USA). The 3′-UTR reporter vectors for human *ROCK1*, *WNT7A*, *KNP4A*, *PDE4B* and *VEGF-A* were purchased from OriGene (Rockville, MD, USA). A luciferase reporter assay was performed as described previously [9]. In brief, EC cells were cotransfected with the reporter plasmid and miR-361 mimic, miR-361 inhibitor or corresponding negative control (Ambion, Austin, TX, USA). After 48 h, luciferase activity was measured with the Dual-Luciferase Reporter Assay System (Promega, Madison, WI, USA). Firefly luciferase activity was normalized to Renilla luciferase activity for each sample.

### RNA immunoprecipitation assay

An RNA immunoprecipitation (RIP) assay was performed with the Magna RIP RNA-Binding Protein Immunoprecipitation Kit (Millipore, Bedford, MA, USA) following the manufacturer’s protocol. Briefly, EC cells were transfected with miR-361 mimic or control mimic. After 48 h, the EC cells were collected and lysed using RIP lysis buffer. An anti-Argonaute2 (Ago2) antibody (Millipore, MA, USA) or negative control normal mouse IgG (Millipore, MA, USA) was conjugated to magnetic beads and incubated with whole cell extract. The immunoprecipitated RNAs were isolated, and a qRT-PCR assay was used to detect the expression of NEAT1.

### Western blot analysis

Cells were lysed with M-Per Mammalian Protein Extraction Reagent (Pierce, Rockford, IL). Equal amounts of the extracts were loaded, subjected to 10% SDS-PAGE, transferred onto nitrocellulose membranes, and probed by antibodies against STAT3 (Cell Signaling), E-cadherin (Cell Signaling), CD133 (Abcam), MEF2D (Abcam), ROCK1 (Abcam), WNT7A (Abcam), KPNA4 (Abcam), VEGF-A (Santa Cruz), Snail (Santa Cruz), Survivin (Santa Cruz) and GAPDH (Santa Cruz) at 4 °C overnight. After incubation with the corresponding secondary antibodies, signals were detected with an ECL detection kit (Amersham Pharmacia Biotech, UK).

### Transient transfection

Expression vectors encoding STAT3, MED2D, ROCK1, WNT7A and KPNA4 and the corresponding empty control vector (OriGene, MD, USA) were transfected using Lipofectamine 2000 (Invitrogen, Carlsbad, CA) according to the manufacturer’s protocol.

### Statistical analysis

The results are shown as the mean ± standard deviation of triplicate experiments. Statistical evaluations were carried out with SPSS 13.0 software (Chicago, IL, USA). Two-tailed Student’s *t*-tests were used to compare the means of two groups, and ANOVA was used to compare the differences among multiple groups. A *P* value < 0.05 was considered statistically significant.

## Results

### Expression of the lncRNA NEAT1 is upregulated in invasive, sphere-forming and TX-resistant HEC-50 cells

**Fig. 1:**
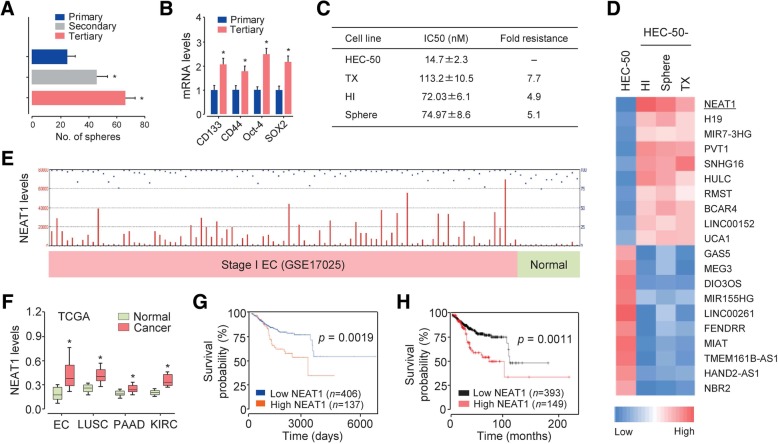
NEAT1 expression is upregulated in invasive, sphere-forming and TX-resistant HEC-50 cells. (**a**) The sphere-forming efficiency of HEC-50 cells was measured in three serial passages. The numbers of primary, secondary (generated from dissociated primary spheres) and tertiary (generated from dissociated secondary spheres) spheres are shown. (**b**) qRT-PCR was used to analyze CSC-related gene expression in primary and tertiary spheres. (**c**) Paclitaxel (TX)-resistant cell lines derived from HEC-50 cells were created as described in the Materials and Methods section. Chemosensitivity data (expressed as the IC50 and fold resistance) for the invasive, sphere-forming and TX-resistant HEC-50 derivatives and parental cells to TX treatment are summarized. (**d**) A heatmap of lncRNA expression shows lncRNAs with upregulated expression (NEAT1, H19, PVT1, UCA1, MIR7-3HG, SNHG16, HULC, RMST, BCAR4 and LINC00152) or downregulated expression (MEG3, GAS5, DIO3OS, MIR155HG, LINC00261, FENDRR, MIAT, TMEM161B-AS1, HAND2-AS1 and NBR2) in the invasive, sphere-forming and TX-resistant HEC-50 cells compared with that in the parental cells. (**e**) The NCBI Gene Expression Omnibus (GEO) dataset GSE17025 was used for profiling NEAT1 expression in stage I EC samples (*n* = 91) and normal endometrium samples (*n* = 12). (**f**) NEAT1 expression was higher in tissue samples from different types of cancer than in corresponding normal tissue samples from the TCGA database. (**g** and **h**) Kaplan-Meier overall survival analysis was used to assess EC patients with high or low NEAT1 expression based on the TCGA data with the UALCAN web tool (**g**) and KM Plotter (**h**). *P < 0.05

To identify the lncRNAs that govern invasion, sphere formation and TX resistance in aggressive EC, we established HEC-50 human EC cell derivatives that display a high invasive ability as measured by Matrigel penetration [8], exhibit enhanced self-renewal ability and CSC-related gene overexpression (CD133, CD44, Oct-4 and SOX2) (Fig. 1a-b) or are resistant to TX treatment (Fig. 1c). Interestingly, the invasive and sphere-forming cell derivatives were also more resistant to TX treatment than the parental HEC-50 cells (Fig. 1c).

LncRNA microarray profiling was performed to identify lncRNAs with differential expression between the parental cells and their derivatives. Ten lncRNAs, including known oncogenic lncRNAs involved in EC (H19 [6], PVT1 [13] and UCA1 [14]), NEAT1, MIR7-3HG, SNHG16, HULC, RMST, BCAR4, and LINC00152, exhibited higher expression, while 10 lncRNAs including MEG3 and GAS5, which have tumor suppressor roles in EC [6], and DIO3OS, MIR155HG, LINC00261, FENDRR, MIAT, TMEM161B-AS1, HAND2-AS1, and NBR2 had lower expression in the invasive, sphere-forming and TX-resistant HEC-50 derivatives than in the parental cells (Fig. 1d). We subsequently used a qRT-PCR assay to validate the differential expression of these 20 lncRNAs (data not shown). We chose to focus on NEAT1, whose expression was the most increased in all derivatives compared with that in the parental cells.

We compared NEAT1 levels between normal endometrial tissue and stage I EC tissue using the human microarray dataset GSE17025 downloaded from the NCBI Gene Expression Omnibus (GEO) database. NEAT1 was highly expressed in EC tissue samples but rarely expressed in normal endometrium samples (Fig. 1e). When analyzing NEAT1 expression in different types of cancer using The Cancer Genome Atlas (TCGA) data from the MethHC database (http://methhc.mbc.nctu.edu.tw/php/index.php), NEAT1 levels were found to be significantly higher in lung, pancreatic and kidney cancer and EC samples than in normal tissue samples (Fig. 1f). To assess the association between NEAT1 expression and overall survival in patients with EC, we used the UALCAN web tool (http://ualcan.path.uab.edu/index.html) and KM Plotter (http://kmplot.com/analysis/), which integrated gene expression and clinical data from EC patients and divided the patients into a NEAT1-high group and a NEAT1-low group. Overall survival was relatively lower in the NEAT1-high group than in the NEAT1-low group (Fig. 1g-h). These results indicated that NEAT1 may positively regulate the occurrence and development of cancer invasion, CSC-like properties and chemoresistance in aggressive EC.

### NEAT1 enhances invasive and sphere-forming capabilities and confers TX chemoresistance in aggressive EC cells

**Fig. 2:**
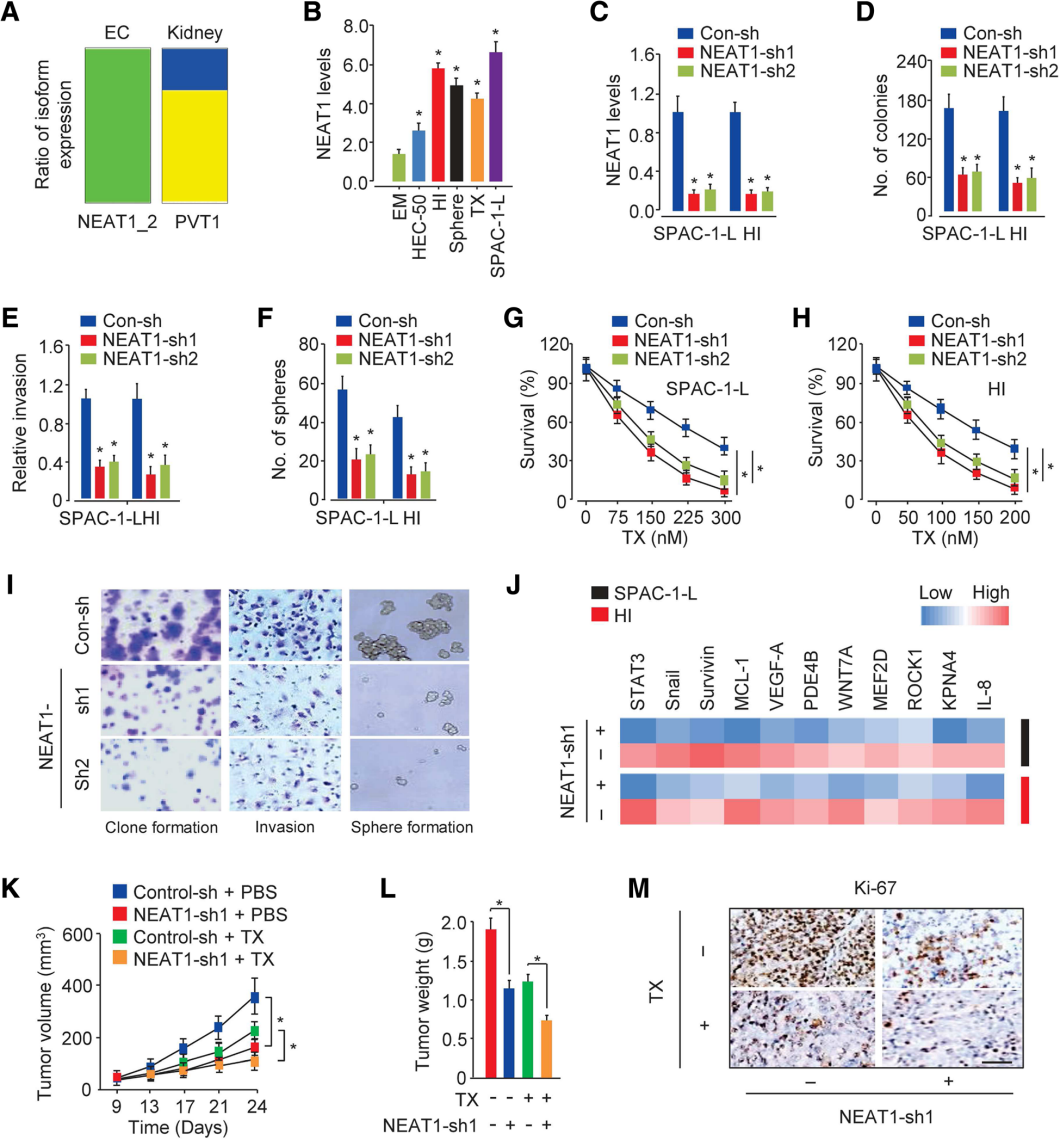
NEAT1 enhances invasive and sphere-forming capabilities and confers paclitaxel chemoresistance in aggressive EC cells. (**a**) RNA sequencing was used to measure the expression levels of one NEAT1 isoform across 545 TCGA EC patient samples and two PVT1 isoforms across 290 TCGA kidney cancer patient samples obtained from the ISOexpresso platform. Each color represents a specific isoform. (**b**) qRT-PCR was used to analyze NEAT1 expression in the normal endometrial epithelial cell line EM, the serous EC cell line SPAC-1-L, invasive HI cells, sphere-forming HEC-50 cells, TX-resistant HEC-50 cells and parental HEC-50 cells. (**c**) The efficiency of NEAT1 expression knockdown in SPAC-1-L and HI cells by two shRNAs was verified using qRT-PCR analysis. (**d**-**f**) The effects of NEAT1 expression knockdown on cell colony formation (**d**), cell invasion (**e**) and sphere formation (**f**) were examined using colony formation, Matrigel invasion and sphere formation assays, respectively, in SPAC-1-L and HI cells. (**g**, **h**) Cell survival was examined by a cell viability assay in SPAC-1-L (**g**) and HI (**h**) cells transfected with or without NEAT1-specific shRNAs and treated with TX. (**i**) Representative images from colony formation, invasion and sphere formation assays using HI cells transfected with or without NEAT1-specific shRNAs are shown. (**j**) A heat map of qRT-PCR data shows the expression of the indicated genes in SPAC-1-L and HI cells transfected with or without NEAT1-specific shRNAs. (K, L) Growth curves (**k**) and quantification of the weight (**l**) of subcutaneous control or NEAT1-silenced SPAC-1-L xenografts treated for 24 days with vehicle or TX are shown. (**m**) Representative IHC images of Ki-67 expression in tumors from the mice described in K and L are shown. Scale bar: 50 μm. *P < 0.05

NEAT1 encodes two variants: NEAT1_1 (3,756 bp) and NEAT1_2 (22,743 bp) [7]. We examined RNA expression data from the ISOexpresso platform, a web-based tool for isoform-level expression analysis in human cancer (http://wiki.tgilab.org/ISOexpresso/), and we found that the longer variant of NEAT1 (NEAT1_2), rather than NEAT1_1, was expressed in EC tissue (Fig. 2a). Moreover, we observed two PVT1 variants in kidney cancers (Fig. 2a), as reported previously [15]. Therefore, we decided to further characterize NEAT1_2 to determine its role in aggressive EC progression.

We validated the expression of NEAT1 in the normal endometrial epithelial cell line EM and in parental HEC-50 cells and their derivatives using qRT-PCR analysis. NEAT1 expression was higher in EC cells than in EM cells, and SPAC-1-L and HI cells exhibited higher levels of NEAT1 than the other EC cells (Fig. 2b). To investigate whether NEAT1 has critical roles in promoting invasion, sphere formation and TX resistance in aggressive EC cells, we stably knocked down the expression of NEAT1 in SPAC-1-L and HI cells using NEAT1-specific shRNAs (Fig. 2c). Compared with the control-transfected cells, the SPAC-1-L and HI cells transfected with NEAT1-specific shRNAs formed fewer colonies, were less invasive, lost their sphere-forming capacity and were more sensitive to TX treatment (Fig. 2d-i). To determine whether NEAT1 acts as a promoter of tumor progression and chemoresistance in vivo, we established subcutaneous tumors in nude mice using the SPAC-1-L cells with NEAT1 expression knockdown or control cells transfected with a control shRNA plasmid. In the absence of TX treatment, NEAT1 expression knockdown inhibited xenograft growth (Fig. 2k-l). However, the combination of NEAT1 expression knockdown and TX treatment resulted in more pronounced tumor growth suppression than TX treatment or NEAT1 expression knockdown alone, as evidenced by significant reductions in tumor volume and tumor weight (Fig. 2k-l). Consistent with our in vivo data, the IHC analysis data demonstrated that compared to NEAT1 expression knockdown or TX treatment alone, silencing NEAT1 combined with TX treatment significantly suppressed the expression of the cell proliferation marker Ki-67 (Fig. 2m). Thus, NEAT1 enhanced invasive and sphere-forming abilities and was required for TX tolerance in aggressive EC cells.

### NEAT1 functions as a molecular sponge for miR-361 in aggressive EC cells

**Fig. 3:**
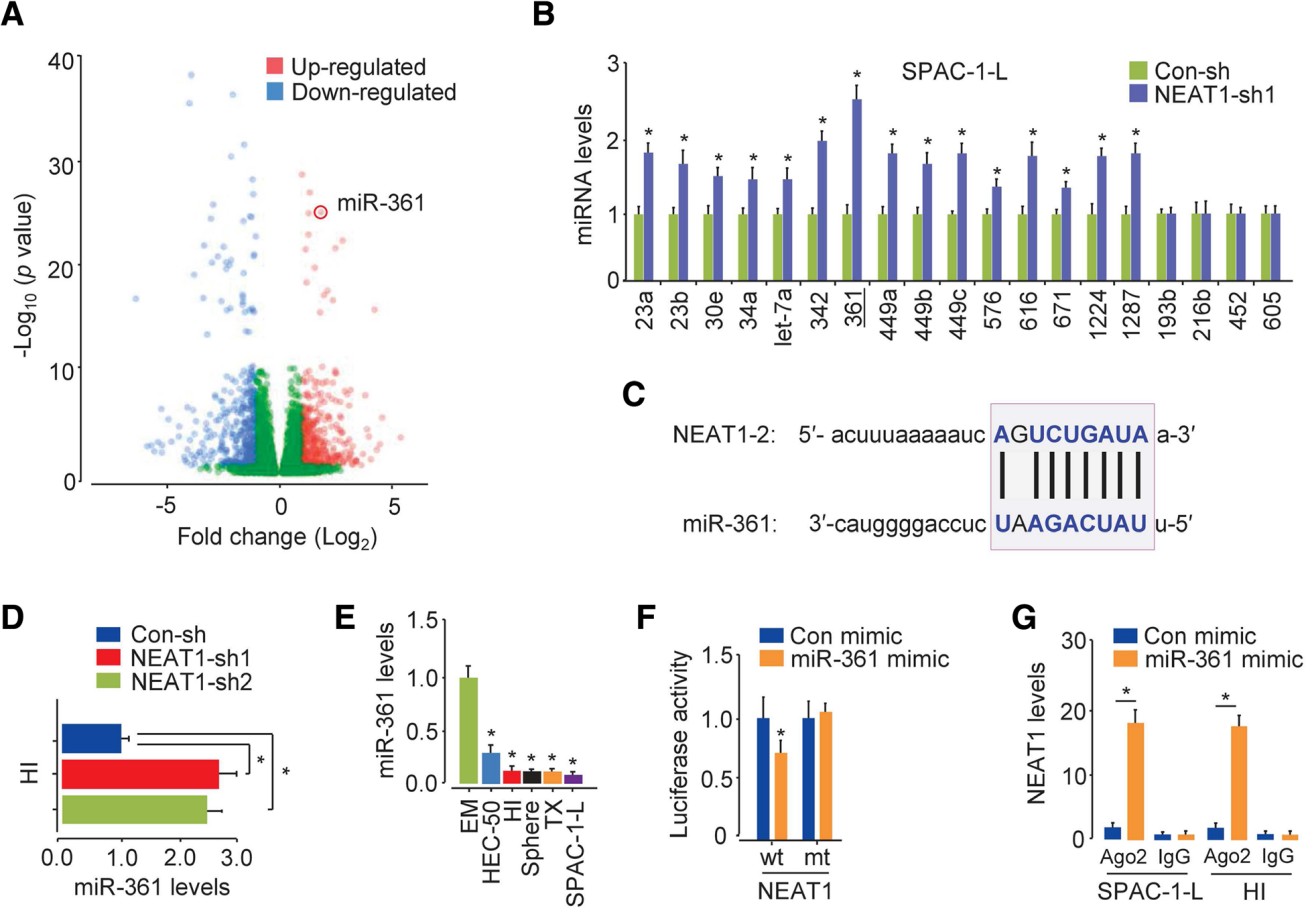
NEAT1 functions as a sponge for miR-361 in aggressive EC cells. (**a**) A volcano plot comparing the expression of miRNAs in SPAC-1-L cells transfected with or without NEAT1-specific shRNAs. (**b**) qRT-PCR analysis of the indicated miRNAs in SPAC-1-L cells transfected with or without NEAT1-specific shRNAs. (**c**) The miR-361 binding sequence in the NEAT1 sequence. (**d**) qRT-PCR analysis of miR-361 expression in control or NEAT1-knockdown SPAC-1-L cells. (**e**) qRT-PCR analysis of miR-361 expression in EM cells, SPAC-1-L cells, invasive HI cells, sphere-forming HEC-50 cells, TX-resistant HEC-50 cells and parental HEC-50 cells. (**f**) Luciferase reporter assay with SPAC-1-L cells cotransfected with a luciferase reporter plasmid containing wild-type (wt) or mutant (mt) NEAT1 and a miR-361 mimic. (**g**) RIP assays performed with SPAC-1-L and HI cells that transiently overexpressed miR-361, followed by qRT-PCR to detect NEAT1 expression associated with Ago2. *P < 0.05

To identify miRNAs that might be regulated by NEAT1, we used a miRNA microarray to compare miRNA levels between the SPAC-1-L cells with NEAT1 expression knockdown and control cells. The expression of a number of miRNAs, including known tumor suppressor miRNAs (let-7a and miR-34a) and miR-361, was markedly upregulated in NEAT1-knockdown SPAC-1-L cells (Fig. 3a). Using qRT-PCR assays, we confirmed that the expression of let-7a, miR-23a/b, miR-30e, miR-34a, miR-342, miR-361, miR-449a/b/c, miR-576, miR-616, miR-671, miR-1224 and miR-1287 was significantly upregulated expression in NEAT1-knockdown SPAC-1-L cells, whereas a selection of other miRNAs (including miR-193b, miR-216b, miR-452 and miR-605) remained unchanged (Fig. 3b). Given that miR-361 was one of the miRNAs with the most upregulated expression in response to NEAT1 expression knockdown, we hypothesized that NEAT1 contributes to metastatic ability and increases chemoresistance by modulating miR-361 and/or other miRNAs.

Using the bioinformatic tool starBase v3.0 (http://starbase.sysu.edu.cn/index.php), we predicted miRNAs with the potential to interact with NEAT1 and identified a putative binding site for miR-361 in the NEAT1 sequence (Fig. 3c). Thus, we reasoned that NEAT1 sponges miR-361 to downregulate miR-361 expression. To test this idea, we performed qRT-PCR assays and found that knocking down NEAT1 expression in HI cells significantly increased miR-361 levels (Fig. 3d). After verifying that miR-361 expression was lower in EC cells than in EM cells (Fig. 3e), we investigated whether NEAT1 can bind to miR-361 in EC cells by constructing luciferase reporter vectors containing WT or mutant NEAT1. The dual-luciferase assays revealed that miR-361 mimic transfection significantly reduced the luciferase activity of WT NEAT1 but had no inhibitory effect on the activity of mutant NEAT1 in SPAC-1-L cells (Fig. 3f). To further validate the interaction between NEAT1 and miR-361, we performed RIP assays with SPAC-1-L and HI cells that transiently overexpressed miR-361. Endogenous NEAT1 was specifically higher in the cells transfected with miR-361 mimic than in the cells transfected with control mimic (Fig. 3g). These data suggested that NEAT1 acts as a sponge to inhibit miR-361 expression in aggressive EC cells.

### NEAT1-mediated miR-361 suppression augments metastatic phenotypes and induces chemoresistance through STAT3 pathway activation

**Fig. 4:**
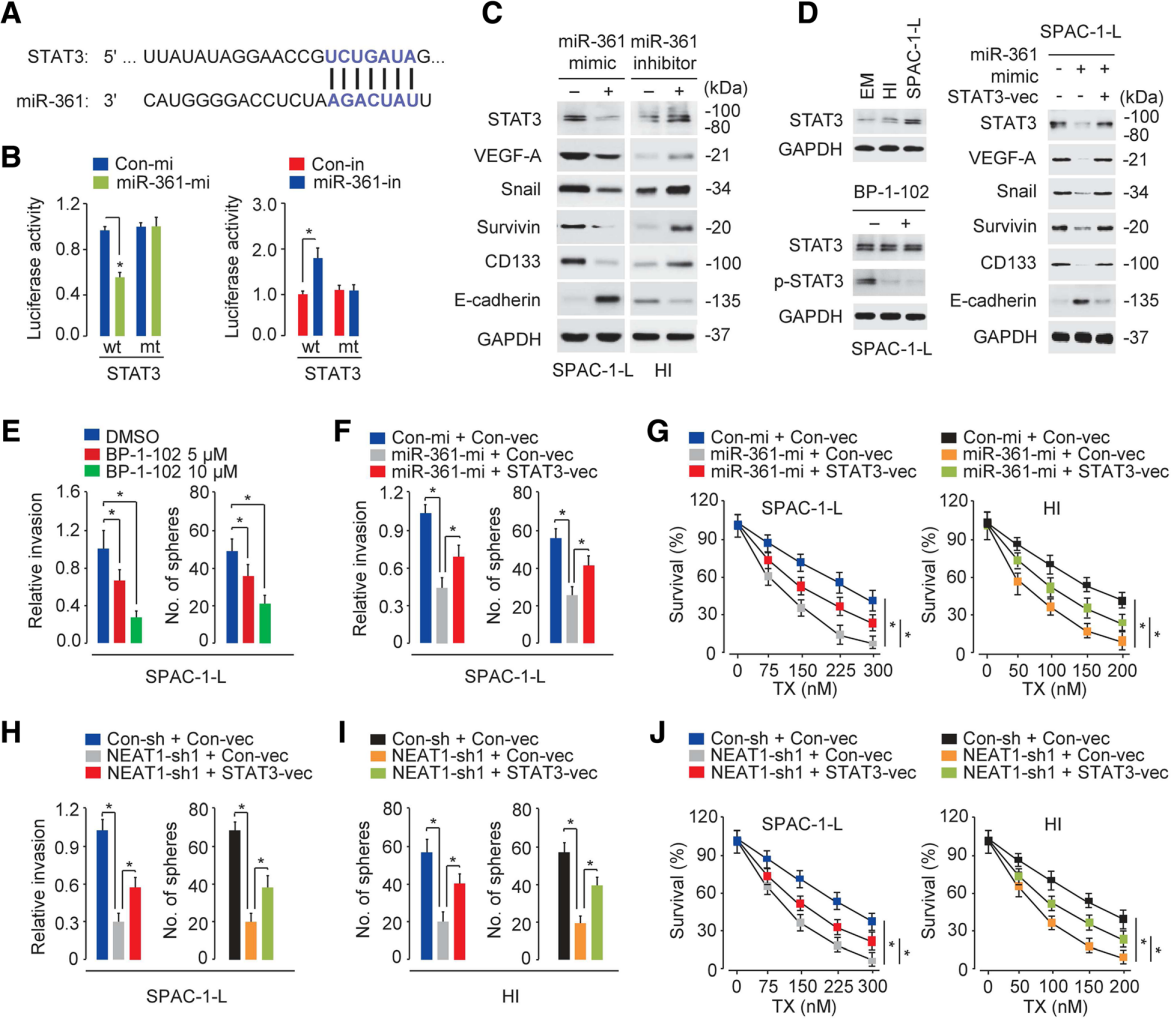
NEAT1-mediated miR-361 suppression augments metastatic phenotypes and induces chemoresistance through STAT3 pathway activation. (**a**) The putative binding site of miR-361 in the *STAT3* 3′-UTR. (**b**) Luciferase reporter assay with SPAC-1-L cells cotransfected with a luciferase reporter plasmid containing wild-type (wt) or mutant (mt) STAT3 and miR-361 mimic (mi) or miR-361 inhibitor (in). (**c**) Western blot analysis of the indicated proteins in SPAC-1-L cells overexpressing miR-361 or HI cells with miR-361 expression knockdown. (**d**) Left panel: The protein expression of STAT3 in EM, SPAC-1-L and HI cells detected using Western blot analysis (upper). The protein expression of STAT3 and p-STAT3 in SPAC-1-L cells treated with or without the STAT3 inhibitor BP-1-102 (bottom). Right panel: Western blot analysis of the indicated proteins in SPAC-1-L cells cotransfected with control or miR-361 mimic with or without a STAT3 expression vector (vec). (**e**) Cell invasion and sphere formation abilities of SPAC-1-L cells treated with or without BP-1-102. (**f**) Cell invasion and sphere formation abilities of SPAC-1-L cells cotransfected with control or miR-361 mimic with or without a STAT3 expression vector. (**g**) Cell survival examined by a cell viability assay in SPAC-1-L and HI cells cotransfected with a control or miR-361 mimic with or without a STAT3 expression vector and treated with TX. (**h**, **i**) Cell invasion, sphere formation and drug sensitivity of control or NEAT1-silenced SPAC-1-L (**h**) and HI (**i**) cells transfected with or without a STAT3 expression vector. (**j**) Cell survival examined by a cell viability assay in control or NEAT1-silenced SPAC-1-L and HI cells transfected with or without a STAT3 expression vector and treated with TX. *P < 0.05

To determine the target genes of miR-361, we used multiple prediction algorithms (TargetScan, microRNA.org and DIANA-MicroT-CDS) and identified a set of common genes targeted by miR-361; these genes included STAT3 (Fig. 4a), which controls diverse hallmarks of human cancer, including proliferation, apoptosis, invasion, CSC generation, metastasis and chemoresistance as well as angiogenesis- or inflammation-associated cancer progression, by regulating the expression of its downstream effectors, such as Snail, MCL-1, Survivin, VEGF-A and IL-8 [16]. Aberrant activation of STAT3 signaling promotes the proliferation and invasion of EC cells derived from both endometrioid and serous ECs [17–19]. According to the reporter assays, the luciferase activity of the WT *STAT3* 3′-UTR was suppressed by miR-361 mimic transfection in SPAC-1-L cells but increased by miR-361 inhibitor transfection in HI cells (Fig. 4b). A mutation in the miR-361 binding site reversed the effects of miR-361 (Fig. 4b). Our western blot analysis indicated that the levels of STAT3, its downstream target genes (including VEGF-A, Snail and Survivin) and the CSC marker CD133 were reduced, but the level of the epithelial marker E-cadherin was increased by miR-361 overexpression in SPAC-1-L cells (Fig. 4c). The inhibition of miR-361 in HI cells caused the opposite effects (Fig. 4c). Consistently, the protein expression of STAT3 was higher in SPAC-1-L and HI cells than in normal EM cells (Fig. 4d, upper panel). Furthermore, blocking STAT3 activity with the STAT3 inhibitor BP-1-102 inhibited STAT3 phosphorylation and inhibited cell invasion and sphere formation in SPAC-1-L cells (Fig. 4d, bottom panel and Fig. 4e), suggesting that STAT3 activation was required for the enhanced invasiveness and CSC-like abilities of aggressive EC cells and that STAT3 was directly targeted by miR-361.

To explore whether the effects of NEAT1 or miR-361 on aggressive EC cells are dependent on the expression of STAT3, we restored STAT3 expression with a STAT3 plasmid vector in miR-361 mimic-transfected SPAC-1-L cells or control cells. The restoration of STAT3 expression eliminated the inhibitory effects of miR-361 overexpression on cell invasion and sphere formation (Fig. 4f). Although overexpression of the miR-361 mimic significantly decreased cell viability during TX treatment, the simultaneous overexpression of STAT3 enhanced TX resistance in SPAC-1-L and HI cells (Fig. 4g). STAT3 overexpression also reversed the NEAT1 expression knockdown-induced inhibition of invasion, sphere formation and TX resistance in SPAC-1-L and HI cells (Fig. 4h-j). Overall, these findings suggested that NEAT1-mediated miR-361 suppression promotes invasion, sphere formation and TX resistance in aggressive EC cells via the activation of the STAT3 pathway.

### MiR-361 downregulates the expression of prometastatic genes and microenvironment-related genes

**Fig. 5:**
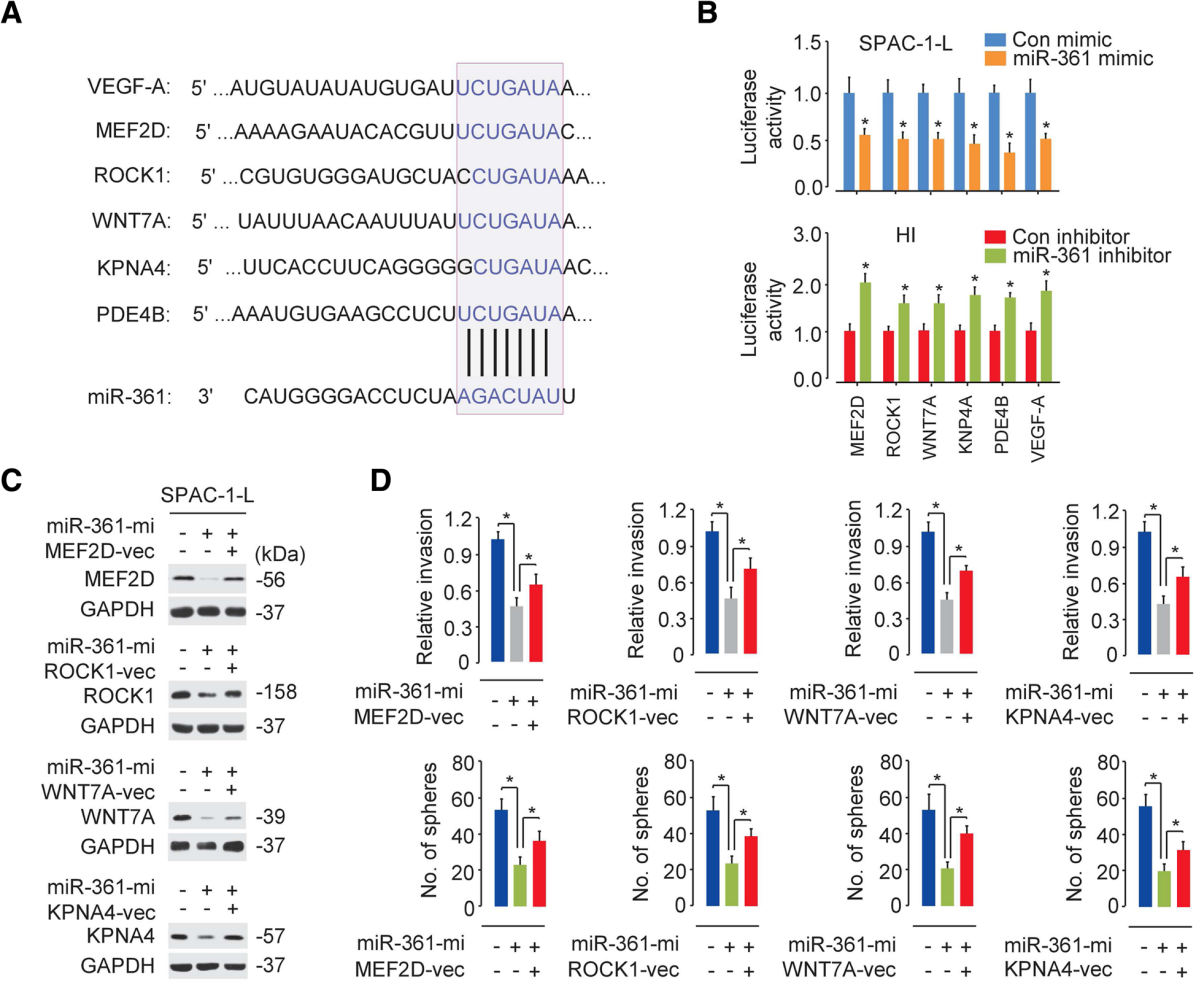
MiR-361 downregulates the expression of prometastatic genes and microenvironment-related genes. (**a**) Predicted interactions between miR-361 and the 3′-UTRs of the indicated genes. (**b**) Luciferase reporter assays using SPAC-1-L cells cotransfected with the indicated luciferase reporters and miR-361 mimic or HI cells cotransfected with the indicated luciferase reporters and miR-361 inhibitor. (**c**) Western blot analysis of the indicated proteins in SPAC-1-L cells cotransfected with control or miR-361 mimic with or without a MEF2D, ROCK1, WNT7A or KPNA4 expression vector. (**d**) Cell invasion and sphere formation abilities of SPAC-1-L cells cotransfected with control or miR-361 mimic with or without an MEF2D, ROCK1, WNT7A or KPNA4 expression vector. *P < 0.05

Using online algorithms, we were able to predict potential miR-361 target genes involved in cancer metastasis, such as *MEF2D* [20], *ROCK1* [21] and W*NT7A* [22], and the tumor microenvironment, including *KPNA4* [23], *PDE4B* [24] and *VEGF-A* (Fig. 5a). To determine whether miR-361 modulates these genes directly, we performed reporter assays by cotransfecting luciferase reporter vectors containing miR-361 binding sites in their 3′-UTRs together with either miR-361 mimic or miR-361 inhibitor. The overexpression of miR-361 significantly reduced the luciferase activities of the WT 3′-UTRs of the indicated genes, while the inhibition of miR-361 significantly increased these activities (Fig. 5b), indicating that these genes were possible targets of miR-361.

**Fig. 6:**
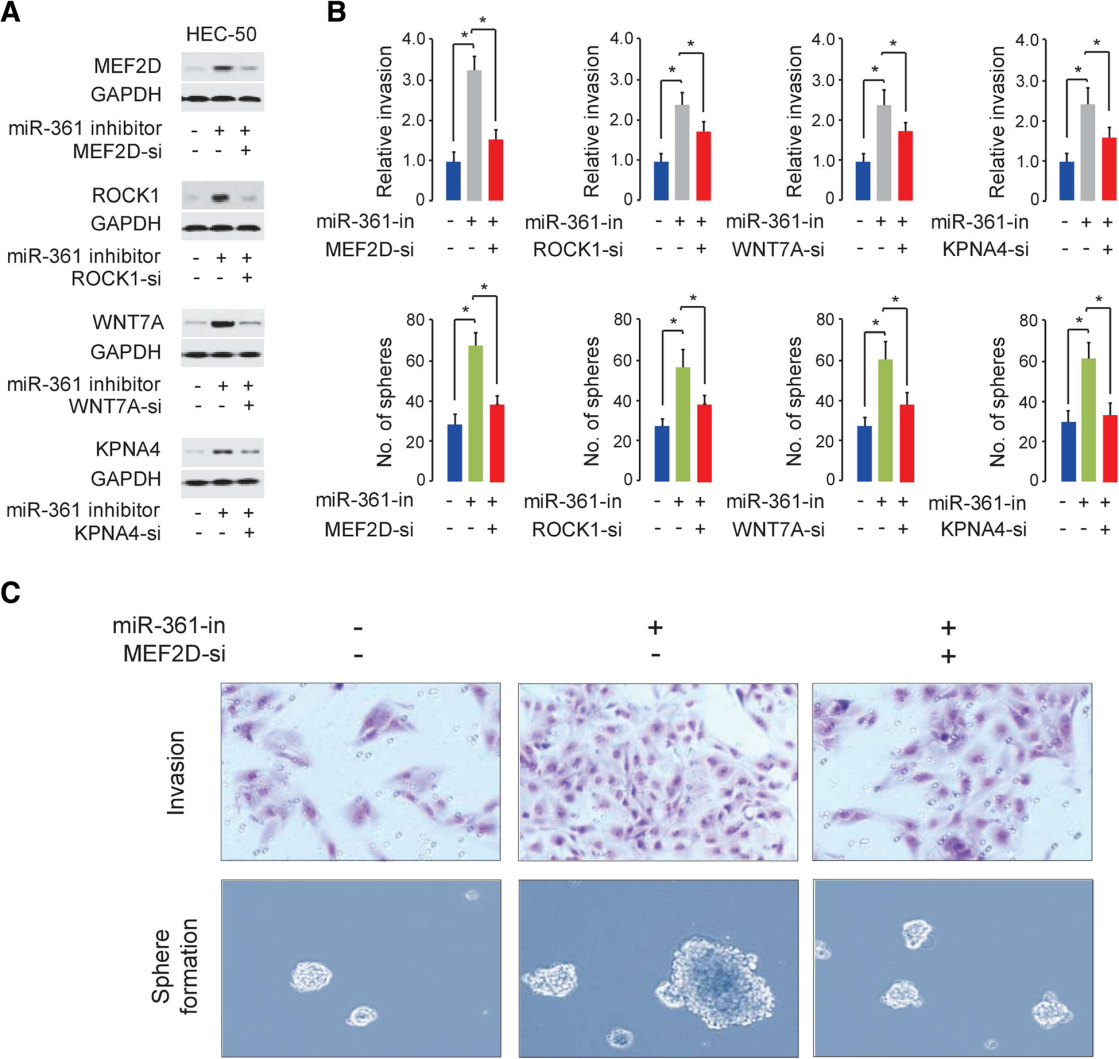
MEF2D, ROCK1, WNT7A and KPNA4 mediate the effects of miR-361 on cell invasion and sphere formation in HEC-50 cells. (**a**) Western blot analysis of the indicated proteins in HEC-50 cells cotransfected with a control or miR-361 inhibitor with or without the indicated siRNA (si). (**b**) Cell invasion (upper) and sphere formation abilities (bottom) of HEC-50 cells cotransfected with a control or miR-361 inhibitor with or without the indicated siRNA. (**c**) Representative images from invasion and sphere formation assays using HEC-50 cells cotransfected with a control or miR-361 inhibitor with or without an MEF2D-specific siRNA. *P < 0.05

We further elucidated whether these genes could modulate the biological functions of miR-361. As expected, the protein levels of MEF2D, ROCK1, WNT7A and KPNA4 as well as cell invasion and sphere formation were decreased when we overexpressed miR-361 in SPAC-1-L cells, and restoring the expression of MEF2D, ROCK1, WNT7A or KPNA4 restored these malignant characteristics in SPAC-1-L cells (Fig. 5c-d). Consistent with these data, silencing MEF2D, ROCK1, WNT7A or KPNA4 expression with a small interfering RNA (siRNA) attenuated miR-361 expression knockdown-induced cell invasion and sphere formation in parental HEC-50 cells, which expresses relatively higher levels of miR-361 (Fig. 3e and Fig. 6). These findings suggest that miR-361 suppresses invasion and sphere formation in aggressive EC cells, at least in part, by regulating the abovementioned prometastatic and microenvironment-related genes.

### MiR-361 mediates the ability of NEAT1 to promote aggressive EC progression

**Fig. 7:**
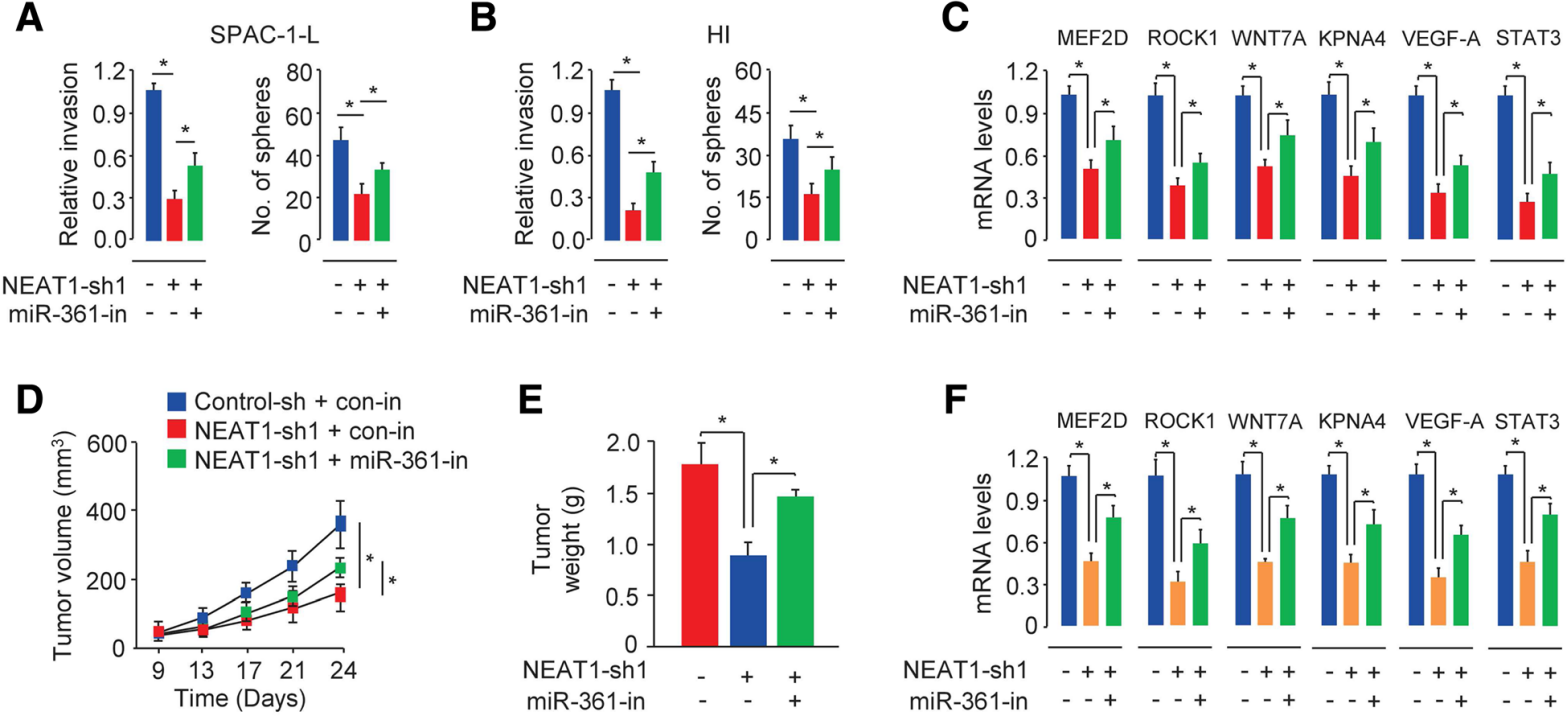
MiR-361 modulates the ability of NEAT1 to promote aggressive EC progression. (**a**, **b**) Cell invasion and sphere formation abilities of control or NEAT1-silenced SPAC-1-L (**a**) and HI (**b**) cells transfected with or without a miR-361 inhibitor. (**c**) qRT-PCR analysis of the indicated miR-361 target genes in control or NEAT1-silenced SPAC-1-L (**a**) and HI (**b**) cells transfected with or without a miR-361 inhibitor. (**d**, **e**) Xenograft tumor formation in nude mice injected with control or NEAT1-silenced SPAC-1-L cells transfected with or without a miR-361 inhibitor. Growth curves (**d**) and tumor weights (**e**). (**f**) Total RNA from tumor tissue analyzed by qRT-PCR assays to determine the mRNA levels of the indicated miR-361 target genes. *P < 0.05

To determine whether NEAT1-induced aggressive phenotypes require the downregulation of miR-361 expression, we knocked down miR-361 expression in SPAC-1-L and HI cells transfected with NEAT1-specific shRNA. The downregulation of NEAT1 expression suppressed cell invasion and sphere formation, and the downregulation of miR-361 by miR-361 inhibitor transfection abrogated the effects of NEAT1 silencing, resulting in increased cell invasion and sphere formation (Fig. 7a-b).

Next, we examined whether NEAT1 modulates the expression of the target genes of miR-361 in aggressive EC cells. Our qRT-PCR assays showed that the expression levels of the miR-361 targets identified here, including *PDE4B*, *WNT7A*, *MEF2D*, *ROCK1*, and *KPNA4*, and several components of the STAT3 pathway, such as *STAT3*, *VEGF-A*, *Snail*, *Survivin*, *MCL-1* and *IL-8*, were downregulated in NEAT1-knockdown SPAC-1-L and HI cells (Fig. 2j). Consistent with these observations, the loss of NEAT1 in SPAC-1-L cells decreased the levels of miR-361 target genes, including *MEF2D*, *ROCK1*, *WNT7A*, *KPNA4*, *STAT3*, and *VEGF-A* (Fig. 7c). This inhibition was partially reversed in SPAC-1-L cells transfected with miR-361 inhibitor (Fig. 7c). Our results suggested that miR-361 mediates the effects of NEAT1 in aggressive EC cells.

Based on the above evidence, we asked whether NEAT1 promotes aggressive EC progression by modulating miR-361 in vivo. We found that miR-361 expression knockdown significantly reversed the NEAT1-specific shRNA-mediated decreases in tumor volume and weight and increased *MEF2D*, *ROCK1*, *WNT7A*, *KPNA4*, *VEGF-A* and *STAT3* levels in mouse tumor samples (Fig. 7d-f), further validating that miR-361 functions downstream of NEAT1 to mediate the functions of NEAT1 in aggressive EC progression.

### Clinical significance of miR-361-regulated genes in EC progression

**Fig. 8:**
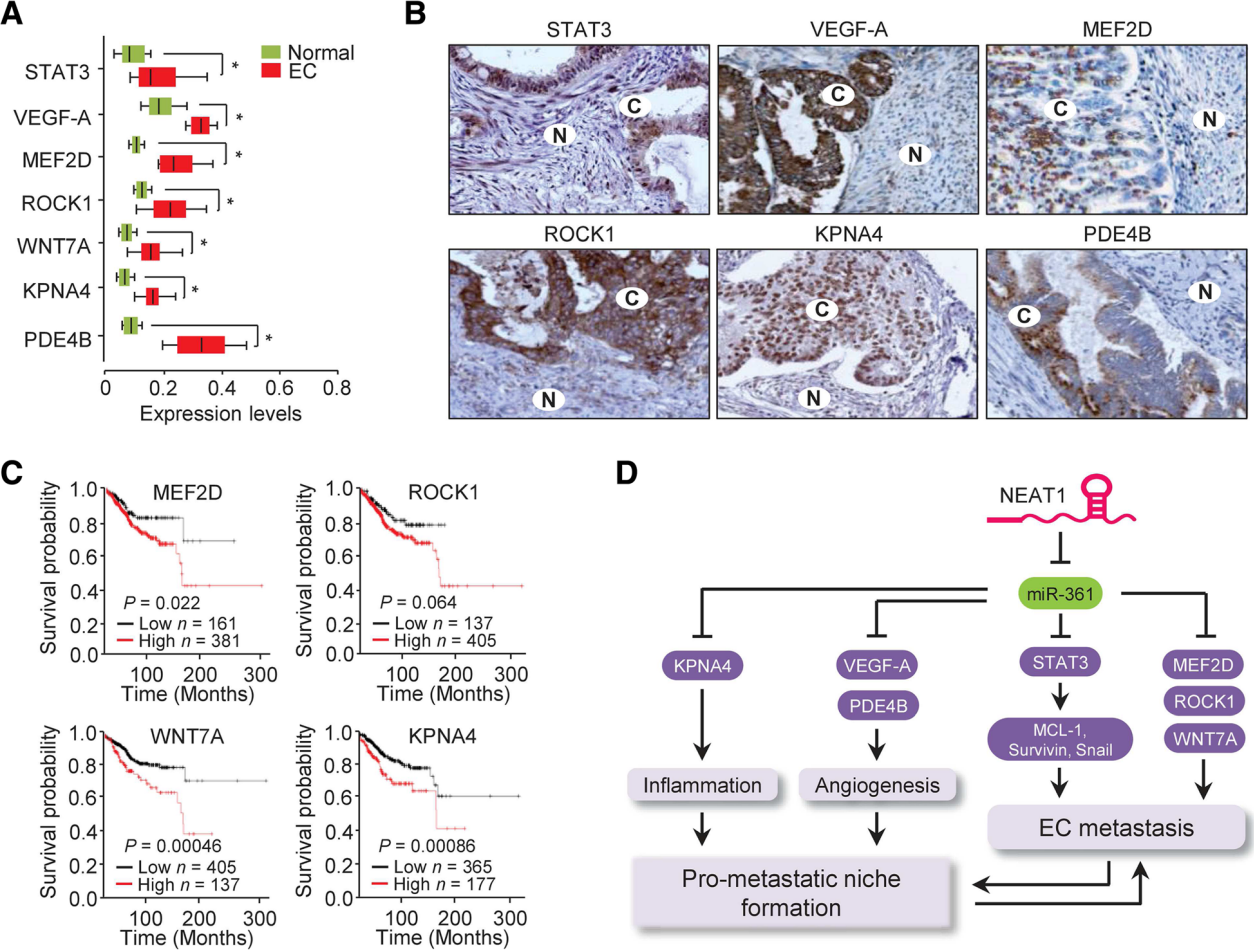
Clinical significance of miR-361-regulated genes in EC progression. (**a**) The mRNA levels of the indicated miR-361 target genes were higher in EC tissue samples than in normal tissue samples in the TCGA data. (**b**) The protein expression of STAT3, VEGF-A, MEF2D, ROCK1, KPNA4, and PDE4D was measured in EC tissue (**c**) and adjacent normal tissue (N). Images were downloaded from the Human Protein Atlas online database. (**c**) Kaplan-Meier overall survival analysis was used to assess EC patients with high or low MEF2D, ROCK1, WNT7A or KPNA4 expression based on the TCGA data from the KM Plotter. (**d**) The proposed mechanisms by which the lncRNA NEAT1 promotes aggressive EC progression via miR-361-mediated gene networks involving STAT3 signaling, prometastatic genes (*MEF2D*, *ROCK1* and W*NT7A*) and tumor microenvironment-related genes (*VEGF-A*, *PDE4B* and *KPNA4*) are shown schematically. *P < 0.05

To examine the clinical importance of the miR-361-mediated signaling pathway in EC samples, we searched for the mRNA expression of miR-361 target genes in the MethHC database. We found that miR-361-regulated genes (*STAT3*, *VEGF-A*, *MEF2D*, *ROCK1*, *WNT7A*, *KPNA4* and *PDE4B*) were significantly overexpressed in TCGA EC samples compared with those in normal samples (Fig. 8a). In addition, we analyzed their protein expression in clinical EC specimens using tissue microarray data from the Human Protein Atlas database (www.proteinatlas.org). STAT3, VEGF-A, MEF2D, ROCK1, KPNA4 and PDE4B were expressed mainly in the nucleus, cytoplasm and membrane of EC cells but rarely or not expressed in adjacent normal tissues (Fig. 8b). Finally, we assessed the associations of MEF2D, ROCK1, WNT7A and KPNA4 expression with the overall survival rate of EC patients using the online survival analysis tool, Kaplan-Meier plotter (http://kmplot.com/analysis/). The patients with high levels of *MEF2D*, *ROCK1*, *WNT7A* or *KPNA4* mRNA displayed shorter overall survival periods (Fig. 8c). Taken together, the results support that these miR-361 target genes could contribute to EC progression.

## Discussion

Aggressive EC tends to invade adjacent normal tissue and be more resistant to chemotherapy [2]. The elucidation of the molecular mechanisms underlying the tumorigenesis, invasion and chemoresistance of aggressive EC is urgently required. In the current study, we demonstrated that NEAT1 sponges miR-361 and suppresses miR-361 expression, thereby activating miR-361-regulated networks involved in invasion, sphere formation and chemoresistance in aggressive EC cells and remodeling the surrounding tumor microenvironment. Mechanistically, NEAT1-induced miR-361 suppression activates the STAT3 pathway, and increases the expression of multiple prometastatic and tumor microenvironment-related genes, which are downstream targets of miR-361 and are critical for the acquisition of metastatic potential and TX treatment resistance in aggressive EC cells (Fig. 8d).

To study the molecular and genetic mechanisms underlying the invasiveness, CSC-like features and TX resistance of aggressive EC, we established subpopulations that all originated from the same EC cell line, HEC-50, but showed differences in invasiveness, sphere formation and drug resistance to TX between the subpopulations and the parental cells. These sublines with different malignant potentials might provide suitable cell models for screening the key molecules involved in the regulation of tumorigenesis and metastasis in aggressive EC.

By comparing the lncRNA expression profiles of these subpopulations with the profile of the parental cell line via a lncRNA microarray assay, we identified a set of lncRNAs, including H19, PVT1, UCA1, MEG3, GAS5, and LINC00261, that promote or suppress EC growth and progression [6, 13, 14, 25] and several candidate lncRNAs, such as SNHG16, with unknown functions in EC. Interestingly, SNHG16, a lncRNA with upregulated expression in invasive, sphere-forming and TX-resistant HEC-50 cells, has been implicated in tumor promotion in a number of cancers other than EC [26, 27]. Detailed studies questioning the significance of these candidate lncRNAs in EC progression should be performed in the near future.

NEAT1 levels were found to be significantly higher in endometrioid EC tissue than in normal endometrial tissue, and higher levels of NEAT1 were positively associated with advanced tumor stage and lymph node metastasis [28]. Our data suggested that NEAT1 was expressed at low levels in the epithelium of normal endometrium tissue but was overexpressed in early-stage EC tissue, which further confirmed that the gain of NEAT1 expression is an early event in EC carcinogenesis and might explain, at least in part, why aggressive EC has a tendency for early metastasis [29].

Metastasis is a complicated process that consists of a series of multiple sequential and interrelated steps that include tumor cell detachment from the primary tumor, extracellular matrix invasion, blood or lymphatic vessel penetration, survival within and exit from the circulation and finally proliferation in a distant organ [30]. Tumor cells actively recruit and transform surrounding normal cells to remodel the tumor microenvironment, which in turn facilitates metastasis [31]. We demonstrated that by reducing the levels of miR-361, NEAT1 performed crucial roles in accelerating several critical steps of metastasis, including proliferation, invasion and cancer stemness-related property acquisition (such as self-renewal and drug resistance) and indirectly increased the expression of miR-361 target genes with known functions in forming of the premetastatic niche characterized by increased angiogenesis and inflammation [32]. Thus, NEAT1 participated in the regulation of a broad range of biological processes in aggressive EC through both cell-intrinsic and cell-extrinsic mechanisms. Our results suggested that knocking down NEAT1 expression with shRNAs markedly inhibited malignant phenotypes in aggressive EC cells and sensitized EC cells to TX treatment. Therefore, targeting NEAT1 alone or in combination with chemotherapy could be a promising therapeutic strategy for aggressive EC.

Among the miR-361 target genes identified in this study, MEF2D is involved in tumor promotion in pancreatic cancer [20], hepatocellular carcinoma [33], osteosarcoma [34], lung cancer [35], gastric cancer [36], colorectal cancer [37], and osteosarcoma [38]. ROCK1 contributes to metastasis in ovarian cancer [39] and osteosarcoma [40]. The expression of WNT7A was higher in EC tissue than in normal endometrial tissue, and increased WNT7A expression has been associated with a high tumor grade and stage, increased depth of myometrial invasion, vascular/lymphatic invasion, lymph node metastasis and worse prognosis in EC [22]. Another study verified the growth-promoting role of WNT7A in the EC cell line Ishikawa, where WNT7A activates the Wnt/β-catenin signaling pathway [41]. PDE4B promotes angiogenesis and tumor growth [24, 42]. KPNA4 acts as an activator of NF-κB signaling, which stimulates cancer cell proliferation and invasion and creates a proinflammatory tumor microenvironment that favors prostate cancer metastasis [23, 43]. Additional studies are warranted to determine the exact mechanisms by which these miR-361 target genes control aggressive EC progression and chemoresistance.

## Conclusions

Our data provide important advances in understanding the functional importance and mechanisms of NEAT1 in driving aggressive EC progression. NEAT1 sponges miR-361 to initiate a prometastatic network involving STAT3 signaling, prometastatic genes and tumor microenvironment-related genes. The components of this regulatory network, including NEAT1, miR-361, STAT3, MEF2D, ROCK1, WNT7A and KPNA4, could be considered potential targets for the development of new treatments for aggressive EC.

## Data Availability

Not applicable.

## Abbreviations

CSC: Cancer stem cell
LncRNA: Long non-coding RNA
miRNA: MicroRNA
